# Aggrecan heterogeneity in articular cartilage from patients with osteoarthritis

**DOI:** 10.1186/s12891-016-0944-8

**Published:** 2016-02-18

**Authors:** John S. Mort, Yeqing Geng, William D. Fisher, Peter J. Roughley

**Affiliations:** Research Unit, Shriners Hospital for Children, 1003, boul. Décarie, Montreal, Quebec H4A 0A9 Canada; Department of Surgery, McGill University, Montreal, Quebec Canada; Division of Orthopaedics, McGill University Health Center, 1650 Cedar Avenue, Montreal, Quebec H3G 1A4 Canada

**Keywords:** Aggrecan, Link protein, Proteolysis, Cartilage, Osteoarthritis

## Abstract

**Background:**

Aggrecan degradation is the hallmark of cartilage degeneration in osteoarthritis (OA), though it is unclear whether a common proteolytic process occurs in all individuals.

**Methods:**

Aggrecan degradation in articular cartilage from the knees of 33 individuals with OA, who were undergoing joint replacement surgery, was studied by immunoblotting of tissue extracts.

**Results:**

Matrix metalloproteinases (MMPs) and aggrecanases are the major proteases involved in aggrecan degradation within the cartilage, though the proportion of aggrecan cleavage attributable to MMPs or aggrecanases was variable between individuals. However, aggrecanases were more associated with the increase in aggrecan loss associated with OA than MMPs. While the extent of aggrecan cleavage was highly variable between individuals, it was greatest in areas of cartilage adjacent to sites of cartilage erosion compared to sites more remote within the same joint. Analysis of link protein shows that in some individuals additional proteolytic mechanisms must also be involved to some extent.

**Conclusions:**

The present studies indicate that there is no one protease, or a fixed combination of proteases, responsible for cartilage degradation in OA. Thus, rather than targeting the individual proteases for OA therapy, directing research to techniques that control global protease generation may be more productive.

## Background

**Fig. 1:**
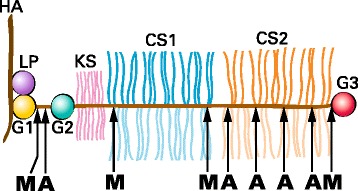
Schematic representation of the structure of aggrecan. Aggrecan is depicted in association with hyaluronan (HA) and link protein (LP) as part of a proteoglycan aggregate. The globular regions of the aggrecan core protein are indicated (G1, G2 and G3) together with the regions of the core protein substituted with keratan sulfate (KS) or chondroitin sulfate (CS1 and CS2) chains. The location of cleavage sites along the core protein for MMPs (M) and aggrecanases (A) are indicated at the bottom of the figure

Aggrecan, the major proteoglycan of articular cartilage, is responsible for the ability of the tissue to withstand the compressive loads that it encounters throughout life. This ability is intimately associated with the structure of aggrecan, in particular its high degree of substitution with sulfated glycosaminoglycan chains and its ability to form large molecular aggregates in association with hyaluronan (HA) [1]. The aggrecan core protein can be divided into discrete structural and functional regions (Fig. 1). At its amino terminus there are two globular regions (G1 and G2) that are separated by a short interglobular domain (IGD). The G2 region is followed by the glycosaminoglycan-attachment region, which consists of a keratan sulfate (KS)-rich domain followed by two chondroitin sulfate-rich domains (CS1 and CS2). At the carboxy terminus is a third globular region (G3) [2]. The G1 region is responsible for the interaction of aggrecan with HA, so preventing free diffusion of the molecule within the tissue. This interaction is stabilized by the presence of a link protein (LP) that non-covalently interacts with both the aggrecan and HA [3]. The KS and CS chains provide the osmotic properties responsible for retaining water under compressive load, so preventing tissue damage [4].

The tissue content and structure of aggrecan does not remain constant throughout life, and its functional ability declines with age [5]. The major detrimental changes are a decrease in aggrecan abundance and a truncation of its core protein. Core protein truncation is due to proteolysis, with each cleavage producing one fragment that remains bound to HA and one that is free. The former fragments are retained within the tissue, whereas the latter are lost by diffusion into the synovial fluid. When cleavage occurs within the IGD or glycosaminoglycan-attachment region, the net result is a decrease in the anionic charge of the aggrecan molecule and a decreased ability to resist compression. Any process associated with increased proteolysis will result in more rapid and extensive aggrecan degradation and loss, weakening the cartilage and ultimately causing tissue erosion [1].

Since, the interglobular domain and the glycosaminoglycan-attachment region of the aggrecan core protein are thought to exist as extended polypeptides [6], they are particularly susceptible to proteolytic attack. In vitro studies of aggrecan degradation have documented cleavage by a diverse variety of proteolytic enzymes. The in vivo situation, however, is dependent on the availability of proteases in the cartilage extracellular matrix. Determination of the amino acid sequence of the termini of aggrecan cleavage products has allowed the production of anti-neo-epitope antibodies specific for the detection of these products in vivo, allowing their characterization in articular cartilage extracts and providing direct evidence for the action of specific proteases [7]. Although a role for the cysteine protease, calpain [8], and the serine protease, HTRA1 [9], has been demonstrated in human articular cartilage, two families of metalloproteases and have been implicated as the major contributors to aggrecan degradation in vivo [10]. Members of the matrix metalloproteinase (MMP) family, in particular stromelysin (MMP3), and MMP13 are produced as proenzymes under inflammatory conditions. Following activation by proteolytic removal of their propeptides, these enzymes cleave aggrecan at sites in the IGD and the CS1/CS2 region. Two members of the ADAMTS (a disintegrin and metalloprotease with thrombospondin motifs) family of metalloproteases, ADAMTS4 and ADAMTS5 which are collectively known as aggrecanases, are also potent aggrecan degrading enzymes [11]. Both of these multidomain enzymes cleave the aggrecan core protein at specific sites in the IGD and the CS2 region. ADAMTS5 has an especially high activity on aggrecan due to the high affinity of its non-catalytic domains for glycosaminoglycan chains [12, 13].

Indirect evidence for the action of proteases in the cleavage of aggrecan has been obtained previously from the characterization of fragments released into the synovial fluid [14–16]. This approach assumes uniform retention of these components in this compartment. The purpose of the current work was to study aggrecan proteolysis within the cartilage from patients with OA, in order to determine how the type and extent of proteolysis varies between individuals.

## Methods

### Source of reagents

Unless otherwise stated, all chemical reagents used in this study were purchased from Sigma.

### Source of OA cartilage

Articular cartilage was obtained from the femoral condyles of patients undergoing total knee replacement (Table 1). Sample collection and analysis protocols (IRB Review Number A03-M26-11A) were approved by the McGill University Institutional Review Board, and informed consent was obtained from each individual. At the time of surgery, all individuals were classified as grade 3-4 based on the Kellgren and Lawrence radiographic scoring system for knee OA [17].

**Table 1:** Details of patients recruited for study

Patient ID	Age	Gender	Condition^†^	Duration (yrs)^a^
1	73	F	OA	12
2	69	F	OA	15
3	74	M	OA	2
4	71	F	OA	3
5	68	F	OA	4
6	61	F	OA	1
7	72	F	OA	3
8	75	F	OA	8
9	66	F	OA	1.5
10	59	F	OA	5
11	51	M	Post-traumatic OA	6
12	89	M	OA	4
13	78	M	OA	1
14	57	F	AVN, secondary OA	8
15	73	F	OA	3
16	59	M	OA	6
17	87	F	OA	1.5
18	89	F	OA	7
19	77	M	OA	1.5
20	77	F	OA	4
21	78	M	OA	5
22	71	F	OA	3
23	71	M	OA	1.5
24	46	F	AVN, secondary OA	4
25	50	F	RA	5.5
26	74	M	OA	1.5
27	69	M	OA	25
28	83	F	OA	3
29	89	F	OA	11
30	72	F	OA second knee	13
31	80	F	OA	20
32	77	M	OA	5
33	71	F	OA	11
34	95	F	OA	3

^†^ *OA* osteoarthritis, *RA* rheumatoid arthritis, *AVN* avascular necrosis

^a^Duration is the time in years between first report of disease symptoms and surgery, for each patient

### Proteoglycan extraction

About 25–30 mg cartilage was obtained from three sites for each femoral condyle: adjacent to the lesion (0–3 mm), remote from the lesion (>6 mm), and from the area midway between the lesion and the remote site (3–6 mm). The cartilage was divided into small pieces and extracted with 20 volumes (v/w) 4 M guanidinium chloride, 100 mM sodium acetate, pH 6.0, containing 1 mM EDTA and a protease inhibitor cocktail (Roche Diagnostics) at 4 °C for 48 h. The extract was recovered by centrifugation and stored at −20 °C.

### Enzyme digestion

Proteoglycan/protein was recovered from the guanidine extracts by precipitation with 9 volumes (v/v) ethanol overnight at −20 °C. The precipitate was recovered by centrifugation and washed two times with 70 % ethanol before drying. The dry pellet was dissolved in 50 mM sodium acetate, pH 6.0, and digested with 0.2 mU/ml keratanase II (Seikagaku) overnight at 37 °C. To adjust the pH, one tenth volume of 1 M Tris HCl /1 M sodium acetate, pH 7.3, was then added and the sample digested with 10 mU/ml chondroitinase ABC (Seikagaku) at 37 °C for 6 h.

### SDS PAGE and immunoblotting

The keratanase/chondroitinase-digested proteoglycan was analyzed on NuPAGE 3–8% Tris-acetate mini gels (Invitrogen) under reducing conditions. Electrophoresis was conducted at 180 V for 1 h, and the proteins then transferred to nitrocellulose membranes (Bio-Rad) by electroblotting at 33 V for 1.5 h. Membranes were probed with rabbit anti-peptide antibodies recognizing the G1, G2, G3 [18, 19] and CS1 [20] regions of aggrecan, or mouse monoclonal antibody 8A4 recognizing link protein [21]. Bound antibody was detected by exposure to a biotinylated anti-rabbit or anti-mouse IgG antibody (Amersham), followed by incubation with Streptavidin-biotinylated horseradish peroxidase (Amersham), and then ECL western blotting detection reagents (Amersham) to visualize reactive bands. The pixel intensity of bands corresponding to free G1 regions generated by aggrecanase or MMP action was quantitated by densitometry and reported to three significant figures (Table 2). The ratio of G1 components generated by aggrecanases and MMPs was calculated.

**Table 2:** Proportions of aggrecanase and MMP G1 degradation products

Patient ID	Aggrecanase G1^a^	MMP G1^a^	Ratio (G1-Agg/G1-MMP)
3	338	290	1.16
4	579	223	2.59
5	251	302	0.83
6	388	165	2.35
7	516	335	1.54
8	456	156	2.92
9	352	352	1.00
10	671	423	1.58
11	904	574	1.57
12	396	139	2.84
13	643	171	3.76
14	226	59.9	3.77
15	263	116	2.26
16	491	190	2.58
17	524	392	1.34
18	503	110	4.57
19	23.2	111	0.21
20	515	200	2.57
21	386	306	1.26
22	452	271	1.66
23	240	177	1.36
24	518	259	2.00
25	218	10.8	20.2
26	323	45.4	7.12
27	405	480	0.84
28	256	280	0.91
29	324	273	1.18
30	726	604	1.20
31	215	96.6	2.23
32	254	243	1.04
33	418	213	1.96
34	56.0	27.2	2.06

^a^Pixel intensity of G1 bands determined by densitometry

### Proteoglycan assay

Proteoglycan recovered by ethanol precipitation and dissolved in the keratanase buffer was assayed for chondroitin sulfate content by the dimethylmethylene blue (DMMB) colorimetric assay using chondroitin 6-sulfate (Sigma) as a standard (12.5 μg/ml to 200 μg/ml) [22]. 20 μl of the proteoglycan sample was combined with 180 μl of DMMB solution (46 mM DMMB (Aldrich), 40 mM glycine, and 40 mM NaCl, pH 3.0) in a 96-well plate, and the absorbance at 530 nm was monitored.

### Reduction and alkylation

Dithiothreitol (5 mM final concentration) was added to 100 μl samples of the guanidine extracts and incubated at 37 °C for 4 h. Iodoacetamide (15 mM final concentration) was then added and incubation continued overnight at room temperature. Samples were then ethanol precipitated as described above and redissolved in 50 mM Tris, pH 7.5.

### Protease digestion

Purified human neonatal aggrecan (4 mg/ml in water) [5] and ethanol-precipitated guanidine extracts were digested with trypsin (10 μg/ml), ADAMTS5 (2 nM) [23] or MMP3 (2 μg/ml; Abcam). Digestion was performed overnight at 37 °C in 50 mM Tris, pH 7.5, containing 1 mM or 5 mM CaCl_2_ (for trypsin or metalloproteases, respectively). The trypsin digestion was terminated by the addition of phenylmethylsulfonylfluoride (PMSF, 1 mM final concentration).

### Agarose gel electrophoresis

Reduced and alkylated proteoglycans were analyzed by electrophoresis on 1.2 % agarose gels [24] for 1 h at 90 V. Proteoglycan (8 μg chondroitin sulfate) was used per well. After electrophoresis, the gel was stained for 1 h using 0.02 % Toluidine Blue and then destained with 3 % acetic acid. In addition, trypsin digests of aggrecan were transferred to a cetylpyridinium chloride-treated nitrocellulose membrane by capillary transfer after electrophoresis [25]. Membranes were probed with an anti-CS1-peptide antibody raised in rabbits against an ovalbumin conjugate of the peptide *G*RIEWPSTPTVGEL*GC* (Uniprot entry P16112, residues 924–936 - italicized residues were added to block the antigenic sequence and to provide a thiol group for coupling to ovalbumin) [20].

### Genomic DNA isolation and sequencing

Genomic DNA was isolated following proteolytic solubilization of cartilage. 50 mg cartilage was digested with 0.5 mg proteinase K in 50 mM Tris HCl, 5 mM EDTA, pH 8.0 at 55 °C for 48 h, and genomic DNA was recovered by precipitation with one volume of isopropanol. The region of the aggrecan gene encompassing the location encoding the epitope recognized by the anti-CS1 antibody was amplified by PCR using the primers GTGGTGACTTCACAGGCAGT and GCCCACTGAGGTCTCCTACT. PCR products were then sequenced at the McGill University Genome Quebec core facility.

### Histology and immunohistochemistry

Full thickness cartilage was fixed in periodate-lysine-paraformaldehyde [26] for 4 h at room temperature, followed by overnight at 4 °C, and then embedded in a mixture of 20 % sucrose/OCT compound (Tissue-Tek). Cryosections were cut at 8 μm and stored at −20 °C. For histology, sections were stained with Safranin O/ Fast green. For immunohistochemistry, sections were treated with 4 % formaldehyde for 10 min, then with chondroitinase ABC (0.25 mU/ml) in the presence of protease inhibitors (PMSF, 1 mM; iodoacetamide, 1 mM; EDTA, 1 mM; and pepstatin A, 10 μg/ml) for 1 h at 37 °C. After treating sections with 0.3 % H_2_O_2_/methanol for 30 min at room temperature, they were exposed to rabbit antipeptide antibodies (anti-G1, anti-G1 MMP [27] and anti-G1 AGG [28], all diluted 1:200). Bound antibody was identified using the Vectastain ABC kit (Vector Laboratories), and visualized with diamino benzaldehyde substrate. Sections were then counterstained with hematoxylin. Between each step in the procedure, sections were washed 3 times with PBS for 5 min each time.

### Statistics

Pearson product-moment correlation coefficients (r) and non-directional p values were determined to investigate possible correspondences between aggrecan G1 degradation products and either patient age or disease duration.

## Results

**Fig. 2:**
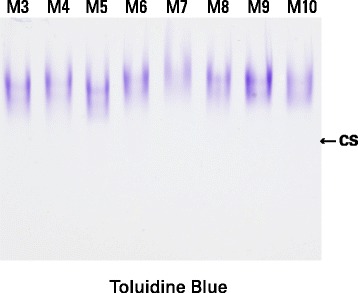
Agarose gel electrophoresis of aggrecan from different individuals. Proteoglycan from guanidine extracts of OA articular cartilage were analyzed by electrophoresis on agarose gels. Aggrecan was visualized by staining the gel with Toluidine blue. Cartilage samples were obtained midway between the lesion and the joint margin from the femoral condyles. Representative samples from eight individuals (M3-M10) are shown. The migration position of isolated chondroitin sulfate chains, indicated as CS, is included to demark the migration position of the smallest possible proteolytic cleavage product

Structural heterogeneity was analyzed in aggrecan isolated from 34 individuals, including 11 males and 23 females, ranging in age from 46 to 89 years at the time of total knee replacement for osteoarthritis (Table 1). The aggrecan was routinely obtained from articular cartilage lying midway between the osteoarthritic lesion and the joint margin. Agarose gel electrophoresis showed size variation in the aggrecan present in different individuals, presumably due to different extents of proteolysis (Fig. 2). However, there was no evidence for extensive degradation producing small fragments of aggrecan bearing only a few CS or KS chains in any individual.

**Fig. 3:**
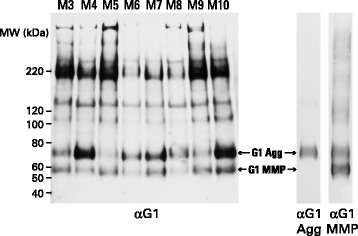
SDS-PAGE and immunoblotting of aggrecan from different individuals. Proteoglycan from guanidine extracts of OA articular cartilage, treated with keratanase and chondroitinase ABC, were analyzed by electrophoresis on polyacrylamide gels, and the fractionated proteoglycan then transferred to nitrocellulose membranes. Aggrecan was visualized by immunoblotting using an antibody recognizing the G1 region. Cartilage samples were obtained midway between the lesion and the joint margin from the femoral condyles and representative samples from eight individuals (M3-M10) are shown. Molecular weights of reference proteins are indicated at the left hand side of the blot. The migration positions of G1 generated by MMP action (G1-MMP) or aggrecanase action (G1-Agg) were determined using anti-neoepitope antibodies specific for their C-terminal peptide sequences (αG1 MMP and αG1 Agg, respectively). αG1MMP shows both terminal neoepitope and some internal sequence recognition

SDS-PAGE analysis of samples treated with keratanase and chondroitinase to remove glycosaminoglycan chains showed fragments of multiple sizes possessing an aggrecan G1 region, ranging from about 60 kDa to over 200 kDa (Fig. 3). Identical fragment sizes were observed in all individuals studied, though the abundance of individual fragments did vary. The two smallest fragments of about 60 kDa (G1 MMP) and 75 kDa (G1 Agg) are indicative of free aggrecan G1 regions resulting from cleavage within the aggrecan IGD by matrix metalloproteinases or aggrecanases, respectively. This was confirmed by immunoblotting using anti-neoepitope antibodies recognizing the new C-terminal amino acid sequences generated by these proteinases (Fig. 3). It is evident that in the majority of individuals the aggrecanase G1 cleavage product predominates (eg individual M4), whereas in others the MMP product is of similar abundance (eg individual M5) or in one case clearly predominates (individual M19; Table 2). These results suggest that the same proteolytic mechanisms were operating in all individuals, but that the contribution from the different proteases involved did vary. No correlation between the extent of aggrecanase or MMP action was observed with either age of the individual (*r* = −0.109 and −0.191, respectively, *p* > > 0.05) or disease duration (*r* = 0.049 and 0.246, respectively, *p* > 0.05) for the group of patients studied. However, on separation of the data for males and females, the abundance of MMP-generated G1 appears to increase with disease duration in the males (*r* = 0.615, *p* = 0.044) (Tables 1 and 2).

**Fig. 4:**
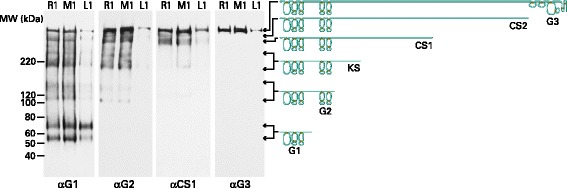
SDS-PAGE and immunoblotting of aggrecan with antibodies to different regions. Proteoglycan from guanidine extracts of OA articular cartilage, treated with keratanase and chondroitinase ABC, was analyzed by electrophoresis on polyacrylamide gels, and the fractionated proteoglycan then transferred to nitrocellulose membranes. Aggrecan was visualized by immunoblotting using antibodies recognizing the G1, G2, CS1 or G3 regions. Cartilage samples were obtained adjacent to the lesion (L), midway between the lesion and the joint margin (M), and remote from the lesion (R) from the femoral condyles. Representative samples from patient 1 are shown. Molecular weights of reference proteins are indicated at the left hand side of the blot. Schematic diagrams depicting the core protein structure of aggrecan fragments of different sizes are included at the right side of the blot

When aggrecan from different sites within the same joint was analyzed by SDS-PAGE, there was no variation in the size range of the fragments observed, but there was variation in the abundance of individual fragments (Fig. 4). The most notable change was in cartilage adjacent to the OA lesion, where fragments larger than the free G1 region were much less abundant than in more remote areas, indicating more extensive degradation. The use of antibodies recognizing different regions of the aggrecan molecule, suggested that the majority of the fragments bear a G1 region and range in size up to that of the intact aggrecan. It appears that heterogeneity is a result of proteolysis within the IGD, surrounding the KS-rich domain, and within the CS2 domain. It also appears that the majority of fragments that no longer possess a G1 domain are lost from the tissue, possibly by diffusion into the synovial fluid due to absence of interaction with HA.

**Fig. 5:**
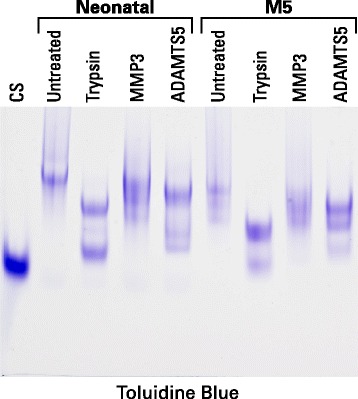
Agarose gel electrophoresis of aggrecan treated with different proteases. Proteoglycan from guanidine extracts of neonatal cartilage and OA articular cartilage (M5) were analyzed by electrophoresis on agarose gels following digestion with trypsin, MMP3 or ADAMTS5. Aggrecan was visualized by staining the gel with Toluidine blue. The OA cartilage samples were obtained midway between the lesion and the joint margin from the femoral condyle (M5). The migration position of isolated chondroitin sulfate chains (CS) is also presented to indicate the migration position of the small proteolytic fragments

To further confirm cleavage within the CS2 domain of aggrecan in OA cartilage, a representative OA sample was further examined by agarose gel electrophoresis following in vitro proteolytic degradation (Fig. 5). Neonatal human aggrecan was used as a control representing intact aggrecan. When the neonatal aggrecan was degraded by trypsin, two fragment sizes were produced, the larger representing the CS1 domain, which is resistant to trypsin cleavage, and the smaller representing fragments from the KS-rich and CS2 domains [29]. ADAMTS5 gave a similar fragment pattern, though the two fragment pools were of larger size. This results from aggrecanases cleaving only within the IGD and CS2 domains. MMP3 shows no extensive cleavage of the aggrecan within its CS1 or CS2 domains. When the aggrecan from the adult OA cartilage was degraded by trypsin, both fragment pools were of smaller size than with the neonatal aggrecan due to the smaller length of the CS chains that are present in the adult [5]. However, the ratio of the two pools was also different with fragments representing the CS2 domain being less abundant than those representing the CS1 domain. This supports proteolysis within the CS2 domain in the OA cartilage, which would result in partial loss from the tissue. This conclusion is reinforced by the ADAMTS5 cleavage pattern, though it should be noted that the CS1-containing fragments now appear as two components due to incomplete cleavage within the CS2 domain in the adult. As with the neonatal aggrecan, MMP3 does not show extensive degradation of the adult aggrecan and is not informative.

**Fig. 6:**
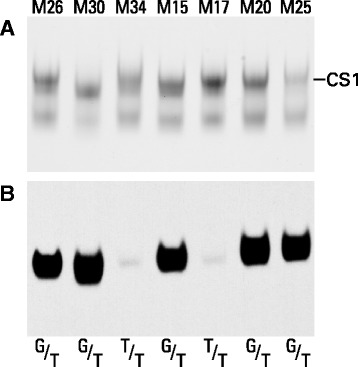
Aggrecan samples from seven osteoarthritis patients were digested exhaustively with trypsin then analyzed by agarose gel electrophoresis. **a** Toluidine Blue staining, **b** immunostaining with an anti-CS1 anti-peptide antibody. The patient identification numbers are indicated above each lane, and the genotypes at position 2789 of the aggrecan cDNA are indicated below each lane

The identity of the CS1 domain following trypsin digestion and analysis by agarose gel electrophoresis was confirmed by immunoblotting. While most samples showed the expected single band coincident with the slower migrating Toluidine Blue staining product, essentially no reaction was seen in the samples from individuals M17 and M34 (Fig. 6). Failure of an anti-peptide antibody to react with its intended epitope can indicate that the sequence of the protein target differs from that used for immunization, for example due to the presence of non-synonymous SNPs. Consultation of the 1000 genomes database indicated that the region used for antibody production contains two known SNPs. While one of these is very rare, the other occurs with nearly equal abundance to the reference sequence isoform. This nucleotide substitution c.2789G > T located in the center of the immunizing peptide replaces a serine residue with an isoleucine, which represents a non-conservative change in sidechain character. Sequencing of genomic DNA showed that most of the individuals were heterozygous at position 2789. However, the two individuals that failed to react with the anti-CS1 antibody were homozygous for the T (Ile) form (Fig. 6), which was not used for antibody preparation.

**Fig. 7:**
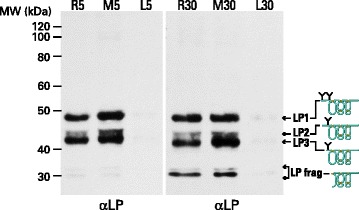
SDS-PAGE and immunoblotting of link protein from different individuals. Proteins from guanidine extracts of OA articular cartilage were analyzed by electrophoresis on polyacrylamide gels, and the fractionated proteins then transferred to nitrocellulose membranes. The membrane was analyzed by immunoblotting using an antibody recognizing link protein (αLP). Cartilage samples were obtained adjacent to the lesion (L5 and L30). midway between the lesion and the joint margin (M5 and M30), and remote from the lesion (R5 and R30) from the femoral condyles and are shown for two representative individuals. Molecular weights of reference proteins are indicated at the left hand side of the blot. Schematic diagrams depicting the structure of link protein components of different sizes are included at the right side of the blot. LP1 and LP2 represent the intact link protein, whereas LP3 and LP frag represent proteolytic cleavage products

Proteolysis was also studied by SDS-PAGE analysis of the link protein that stabilizes the interaction of aggrecan with HA. Different patterns of link protein cleavage were observed depending on the extent of link protein fragmentation. Three major link protein components were evident (LP1, LP2 and LP3). LP1 and LP2 represent the intact link protein and differ only in their degree of N-linked glycosylation, whereas LP3 is generated by MMP action [30]. In some individuals only these three components were present, however, many individuals also showed the presence of LP fragments of smaller size, compatible with cleavage within the disulfide-bonded A-loop. This region is not cleaved by MMPs or aggrecanases, and therefore is indicative of an additional proteolytic mechanism being operative in some individuals. Representative examples of this variation are shown in Fig. 7.

**Fig. 8:**
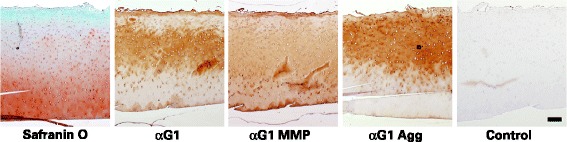
Histology and immunohistochemistry of cartilage. Full thickness articular cartilage was used to prepare frozen sections. Aggrecan in the sections was visualized either by staining with Safranin O or by exposure to antibodies recognizing the G1 region (αG1) or the C-terminal neoepitopes generated in the IGD by the action of MMPs (αG1 MMP) or aggrecanases (αG1 Agg). Control sections in which the primary antibody was replaced by non-immune IgG were also prepared. Representative sections close to the lesion derived from patient 30 are shown. The scale bar indicates 200 μm

To determine whether MMPs and aggrecanases are acting at the same sites within the cartilage, tissue close to the lesion, but with an intact articular surface, was examined by Safranin O staining and immunohistochemistry (Fig. 8). Safranin O staining reveals the sites at which proteoglycan loss is most abundant, and shows that loss increases from the deep layers towards the articular surface. The presence of aggrecan G1 domains also increases towards the surface, and this correlates with the presence of free G1 domains generated by aggrecanase action (corresponding to reactivity to anti-G1Agg). In contrast, MMP-derived G1 domains (corresponding to reactivity to anti-G1MMP) appear more uniformly distributed throughout the cartilage depth. Thus the aggrecan loss typifying the OA cartilage is more associated with aggrecanase than MMP action.

## Discussion

The current work demonstrates that MMPs and aggrecanases are the major proteases involved in aggrecan degradation in adult cartilage, with the aggrecanases being most associated with the increase in aggrecan loss associated with OA. The extent of aggrecan cleavage was highly variable between individuals, but was greatest in areas of cartilage adjacent to sites of cartilage erosion compared to sites more remote within the same joint. The proportion of aggrecan cleavage attributable to MMPs or aggrecanases was also variable, but aggrecanase action was usually predominant in late stage OA as in no case was all the G1 product observed to be converted into the MMP-generated form. Analysis of link protein shows that in some individuals additional proteolytic mechanisms must also be involved to some extent.

There are several plausible mechanisms that could give rise to the individual variation in proteolysis. First, the duration of disease. Even if there is no variation in the abundance of MMPs and aggrecanases produced within the cartilage, a longer time of exposure will always result in a relative increase in MMP-derived G1 regions, as the MMP cleavage site within the IGD is closer to the G1 region than that of the aggrecanases [23]. Second, the presence of joint inflammation. Synovitis will result in cytokine-mediated production of both MMPs and aggrecanases, which can diffuse through the synovial fluid into the cartilage. However, at least in vitro the two major inflammatory cytokines, interleukin 1 and tumor necrosis factor α, stimulate the secretion of active aggrecanases but latent MMPs [31]. While MMP activation may not occur in vivo in all individuals, cytokine release during synovitis will be associated with increased aggrecanase-mediated aggrecan degradation in the more superficial regions of the articular cartilage. Third, the extent of joint loading. The incidence of OA is associated with obesity [32] which will result in increased compressive loading of the cartilage. Increased compressive loading has in turn been associated with increased proteolysis, and at least in the case of the intervertebral disc such compression-mediated proteolysis is associated with MMP action [33]. Thus it is not surprising that there is no unique mechanism responsible for cartilage destruction in OA.

The lack of CS1 epitope recognition in some individuals provides a strong caveat regarding the choice of peptide sequences for antibody production, indicating that naturally occurring polymorphisms need to be taken into account. SNPs are very common, particularly in outbred organisms such as humans. For example, in the human aggrecan cDNA sequence, encoding about 2400 amino acid residues, over 500 SNPs generating missense variants have been reported, of which 50 have minor allele frequencies of greater than 0.1 (http://www.ncbi.nlm.nih.gov/SNP/). These would not be considered when using database cDNA sequences that are usually obtained from single individuals, and which may not be the most representative of the general population. Therefore, when using antibodies recognizing a single peptide sequence, such as anti-peptide or monoclonal antibodies, it is essential to ensure that the target sequence does not include sites of amino acid variation. If this cannot be verified, negative results obtained upon using the antibody must be treated with caution. This is of particular concern when employing commercial antibodies where the epitope recognized by the antibody is often not disclosed or custom antibodies that were prepared prior to the availability of the SNP database. In addition, such single residue substitutions may also modify susceptibility to proteolytic degradation by rendering the protein sequence more (or less) predisposed to the selectivity of proteases present in the tissue.

Previous work relating to aggrecan degradation in OA has centered on the composition of aggrecan fragments that accumulate in patient synovial fluid [14–16, 34]. Using anti-neoepitope antibodies, fragments have been assigned to be products of MMP or aggrecanase action. It was concluded that MMPs are responsible for normal aggrecan turnover whereas aggrecanases contribute to its degradation under pathological conditions [15]. However, when studying aggrecan fragments in synovial fluid, it is not possible to be sure whether degradation generating the fragments occurred within the cartilage or within the synovial fluid itself due to proteases released by the synovium. Our findings on the aggrecan components retained in the cartilage matrix complement these previous findings by showing only what is occurring in the cartilage.

Link protein cleavage can also serve as a monitor of protease action in cartilage in vivo [35]. Previous studies have demonstrated that MMPs are responsible for the generation of LP3 from either LP1 or LP2 [30] and that link protein is resistant to aggrecanase cleavage [19]. In previous studies, the production of link protein fragments has been limited to the action of free radicals or the cysteine proteases cathepsin L, which can cleave within the A-loop of the link protein [35]. Recent studies have shown that cathepsin K can also cleave in this region (unpublished observation). This is of interest as cathepsin K has also been implicated in collagen degradation in arthritic cartilage [36], and could play a more widespread role in extracellular matrix degradation.

## Conclusions

For many years the development of protease inhibitors as therapeutics to slow down or even prevent the cartilage destruction associated with OA has been the goal of arthritis research [37]. The present studies indicate that there is no one protease, or a fixed combination of proteases, responsible for cartilage degradation in OA. Thus, rather than targeting the individual proteases for OA therapy, directing research to techniques that control global protease generation may be more productive [38].

## Abbreviations

ADAMTS: a disintegrin and metalloprotease with thrombospondin motifs
AVN: avascular necrosis
CS: chondroitin sulfate
CS1, CS2: chondroitin sulfate-rich domains of aggrecan
DMMB: dimethylmethylene blue
G1, G2, G3: globular regions of aggrecan
HA: hyaluronan
HTRA1: high temperature requirement A1
IGD: interglobular domain
KS: keratan sulfate
LP: link protein
MMP: matrix metalloproteinase
OA: osteoarthritis
PMSF: phenylmethylsulfonylfluoride
RA: rheumatoid arthritis
SNP: single nucleotide polymorphism
